# Patient and Patient Group Engagement in Cancer Clinical Trials: A Stakeholder Charter

**DOI:** 10.3390/curroncol28020137

**Published:** 2021-04-08

**Authors:** Stéphanie Michaud, Judy Needham, Stephen Sundquist, Dominique Johnson, Sabrina Hanna, Sharareh Hosseinzadeh, Vatche Bartekian, Patricia Steele, Sarita Benchimol, Nathalie Ross, Barry D. Stein

**Affiliations:** 1 BioCanRx, Ottawa, ON K1H 8L6, Canada; smichaud@biocanrx.com; 2 Canadian Cancer Trials Group, Kingston, ON K7L 3N6, Canada; judy.needham@telus.net; 3 Canadian Cancer Clinical Trials Network (3CTN), Toronto, ON M5G 0A3, Canada; Stephen.Sundquist@oicr.on.ca; 4 McPeak-Sirois Group for Clinical Research in Breast Cancer, Montreal, QC H2Y 2H2, Canada; d.johnson@mcpeaksirois.org; 5 The Cancer Collaborative, Montreal, QC H7W 0C3, Canada; sabrina@cancercolab.ca; 6 Novartis Pharma Canada, Dorval, QC H9S 1A9, Canada; sharareh.hosseinzadeh@novartis.com; 7 Vantage BioTrials, Montreal, QC H4M 2N9, Canada; vatche@vantagebiotrials.com; 8 Colorectal Cancer Canada, Montreal, QC H3G 1J1, Canada; patricias@colorectalcancercanada.com (P.S.); saritab@colorectalcancercanada.com (S.B.); 9 Nathalie Ross, Laval, QC H7L 4Z3, Canada; info@nathalieross.com

**Keywords:** cancer patient groups, Canadian Cancer Clinical Trials, patient centricity, patient engagement, Clinical Trials Transformation Initiative, stakeholder charter, real-world evidence, real-world data

## Abstract

Background—to guide the implementation of patient centricity and engagement in cancer clinical trials (CTs) and to operationalize the Canadianized version of the Clinical Trials Transformation Initiative (C-CTTI) model, the development of a charter was identified by cancer CT stakeholders. Methods—the Canadian Cancer Trial Stakeholder Charter (the Charter) was initiated by Colorectal Cancer Canada (CCC) and developed via the—1—formation of an inclusive working group (WG) that drafted the document using recommendations collected during the development of the C-CTTI model; 2—socialization of the draft Charter to solicit feedback from cancer CT stakeholders, including those who attended the 2019 CCC Conference; and 3—incorporation of stakeholders’ feedback and finalization of the Charter by the WG. Results—the Charter was built around five guiding principles—1—patient centricity; 2—commitment to education and training; 3—collaboration as equal and independent partners in research; 4—transparency and accountability; and 5—high standards in data collection integrity and honesty. These principles led to the Charter’s five tenets, which stipulate stakeholder commitments, aiming to make CTs accessible to all patients, improve the design and implementation of CTs to benefit patients, expand recruitment and retention of patients in CTs, and further advance cancer research and treatment. Conclusions—the Charter is intended to integrate the patient voice into the Canadian cancer CT continuum. The next phases of the C-CTTI model include the adoption and implementation of the Charter, the establishment of a patient group training program, and the development of real-world evidence/real-world data methodologies.

## 1. Introduction

Establishing partnerships with patients and patient groups (PGs) to engage them in all levels of clinical trials (CTs), including in real-world evidence research, is increasingly explored and applied globally [1,2,3,4,5,6,7,8,9,10,11,12,13,14,15,16,17,18,19,20,21,22,23]. Keys to such engagement—also referred to as co-design research, participatory research, patient-oriented research (POR), patient-involved research, patient public involvement, patient participation, co-creative research, citizen science, patient-centric initiative, open science—are the understanding and the manifestation of the concept of patient centricity or patient centeredness [1,3,4,24,25,26,27].

Advantages of involving the end-user groups in the broad research agenda include increased research relevance to both clinicians and patients [1], with positive impacts reported in setting research priorities, developing proposals, recruiting and retaining patients, as well as disseminating results, including societal and ethical benefits [2,4,7,9,10,14,28,29,30,31,32,33]. Guidelines and recommendations are emerging on research partnerships with patients, which support the principles of trust, respect, and co-learning [2,7,13,18,19,22,34]. These include POR training for all team members, tools/resources for successful patient engagement, and value for patient partnerships across various stages of the research cycle.

Co-founded by the US Food and Drug Administration and Duke University, and incepted in 2007 as a model for multi-stakeholder engagement in CTs, the Clinical Trials Transformation Initiative (CTTI) led to a significant commitment to help grow and shape patient engagement [18,35]. CTTI defines CT stakeholders as not only the potential participants to enroll in the study, but also the other individuals whose time and effort are needed to develop and execute the project [36]. CT stakeholders can be grouped under—participants (e.g., patients and families), PGs (e.g., support groups and advocacy groups), providers and clinicians (e.g., investigators and community providers), community (e.g., culture and gender), trial management staff (e.g., data management and statistician), site staff (e.g., principal investigators and study coordinators), allied health staff (e.g., nursing and pharmacy), and others (e.g., regulatory authorities, funders, and contract-research organizations). The CTTI recommendations [37] for specific actions and considerations to improve the design and execution of CTs have become a roadmap for patient engagement strategic planning.

Examples of patient engagement initiatives in Canada include recommendations from a panel of expert Canadian emergency medicine researchers on best practices for the engagement of patients in emergency medicine (EM), with the aim to improve EM research by helping researchers select meaningful outcomes, increase social acceptability of studies, and design knowledge translation strategies that target patients’ needs [19]. Focusing on 12 Canadian teams engaged in community-based primary health research, Kendall et al. [38] reported on the engagement of patients and other stakeholders in different stages of research, frequency and length of engagement, as well as facilitators of engagement. Challenges were identified around communication, time, and finding the appropriate stakeholder to engage with. 

Specifically in cancer CTs in Canada, initiatives to engage patients include the Getting better Outcomes with Chimeric Antigen Receptor T-cell therapy (GO-CART) program, which showed that engaging patients in early phase trials helped in each component of the study, including the development of the study protocol, non-technical summary, interview and survey questions, as well as estimating costs for the early economic analysis [4]. 

Despite developments on patient and PG engagement in health research, including the well-known frameworks of Patient-Centered Outcomes Research Institute (PCORI) in the US, INVOLVE in the UK, and Strategy for Patient-Oriented Research (SPOR) in Canada [3,4,6,23,38,39,40], in practice the prevalence of patient engagement across CTs remains extremely low; less than 1% as reported by Fergusson et al. [15] in a systematic review from May 2011 to June 2016. An unmet need resides in providing tangible tools for CT stakeholders to commit to the principle of patient centricity and patient engagement [5,41,42] as well as empowering patients to fulfil their role specifically in cancer CTs. 

**Figure 1:**
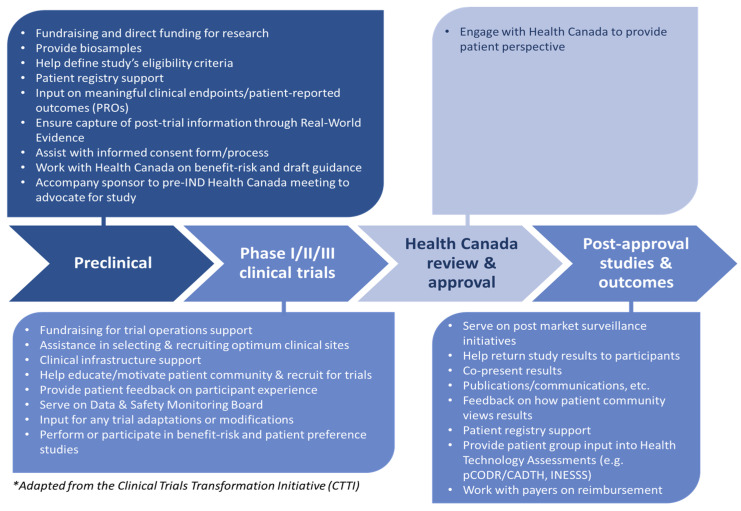
Canadianized Clinical Trials Transformation Initiative (C-CTTI)—a model of a PG pathway for engagement in cancer clinical trials (CTs) in Canada.

Initiated by Colorectal Cancer Canada (CCC) [43] following a consensus meeting in June 2017 on Patient Group Pathway Model to Accessing Cancer CTs, an expert working group (WG) developed a Canadianized version of the CTTI model (C-CTTI), presented in detail in Batist et al. [44] (Figure 1). The C-CTTI takes into account specificities of the Canadian CT environment related to the effects of global decision-making and systems of regulatory and funding approvals. 

As a step to operationalize the C-CTTI model, the CCC and WG were mandated in 2018 to develop a charter that would provide a framework for cancer CTs to help guide the relationships among all stakeholders, empower PGs and patients, make CTs accessible to all patients, improve the design and implementation of CTs to benefit patients, improve recruitment and retention of patients in CTs, and further advance cancer research and treatment. Presented herein are the charter development process, the five tenets that are the pillars to the charter, and the related stakeholders’ commitments. 

## 2. Materials and Methods

### 2.1. Step 1: Drafting the Charter

Following an endorsement of the C-CTTI model [44] in June 2017 as a model to guide patient integration into all phases of cancer research and development in Canada, the participants of the 2018 Patient Group Pathway Model to Accessing Cancer Clinical Trials and Real-World Evidence Conference mandated CCC and the WG (Table S1) to develop a charter document. 

**Figure 2:**
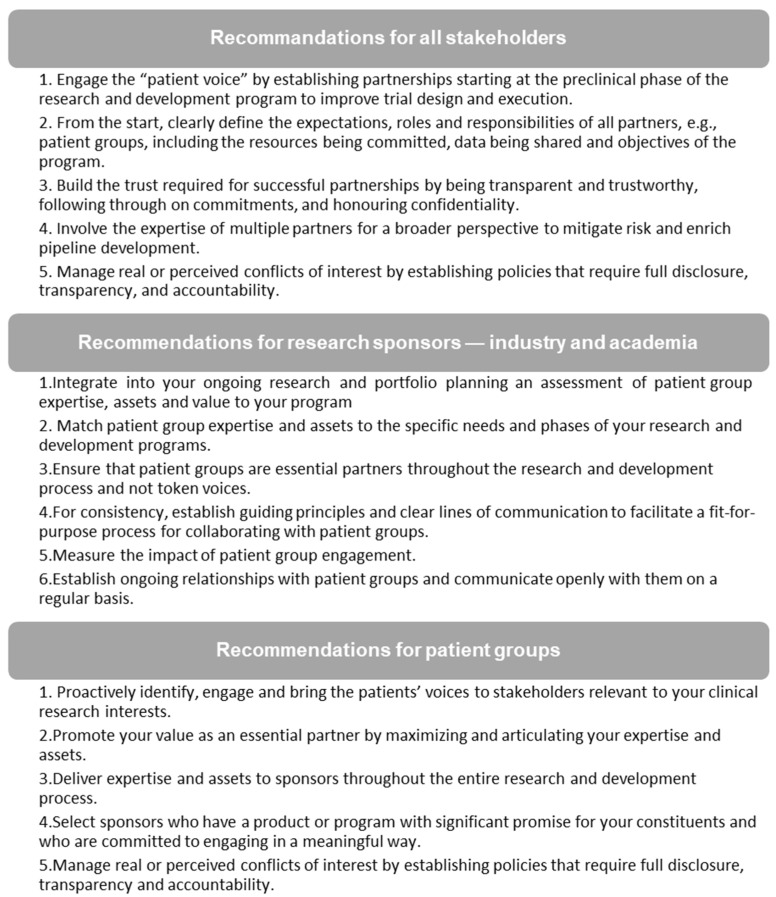
Recommendations from stakeholders of the 2017 Colorectal Cancer Canada (CCC) conference for integrating PGs in cancer CTs, used in the drafting process of the Charter.

Aiming at facilitating the acceptance of PG representatives participating as equal partners in CTs, the WG, composed of scientists/clinicians, individuals from PGs and advocacy groups, cooperative academic CT oncology groups, representatives from health agencies, research networks/consortium, industry partners, contract research organizations (CROs), as well as key participants from CCC, drafted the Canadian Cancer Trial Stakeholder Charter (the Charter). The draft Charter encompassed recommendations collected from the WG consensus meetings (December 2017 and April 2018) during the development of the C-CTTI model (Figure 2). The draft Charter development process included a series of WG sessions, leading to an initial draft considered and revised to integrate feedback, and an updated version made available for a socializing phase in time for the 2019 CCC conference. 

### 2.2. Step 2: Socializing the Charter

In 2018–2019, the draft Charter was circulated to various stakeholders including PGs and trial sponsors, and feedback was incorporated into the different Charter versions. An important source of feedback came from the CCC Patient Group Pathway Model to Accessing Cancer Clinical Trials and Real-World Evidence Methodologies Conference, held in Montreal, Quebec on 16–17 October 2019. During this conference, feedback was collected via a Sprint session [45] using an agile methodology [46]—an iterative approach divided in a series of sub-group discussions and re-group sharing sessions—maximizing the quality and quantity of feedback provided.

The scope of this Sprint session was two-fold—to create awareness for, and educate on, the (new and draft) Charter and to gain stakeholders’ input and feedback with the goal of incorporating this feedback into a final draft of the Charter. The Sprint Backlog, defined as the items to be completed during the Sprint session, were the following five questions:

- How will PGs and patients benefit from this Charter?
- What specific feedback would you like to provide related to wording or information not to be missed?
- What ideas can you provide for successful implementation of this Charter?
- What should the roles and responsibilities of the PG be? and
- What hurdles do you anticipate in the implementation process? What ideas might you have for overcoming these hurdles?

The consolidated feedback from these sub-group discussions and re-group sharing sessions was identified as the minimal viable products (MVPs) from the Sprint session.

### 2.3. Step 3: Finalizing the Charter

The Charter WG reunited after the 2019 CCC meeting to integrate feedback received from the varied stakeholders, specifically to adjust wording for clarity and completeness. The main considerations for finalizing the Charter included—clarifying the intended target of the Charter, identifying a process to create awareness and promote acceptance and trust, developing implementation metrics and measures, as well as addressing privacy considerations. Through a series of wordsmithing iterations, and including the addition of a glossary, the Charter was finalized [47].

## 3. Results

### 3.1. Consolidated Feedback from Stakeholders

**Figure 3:**
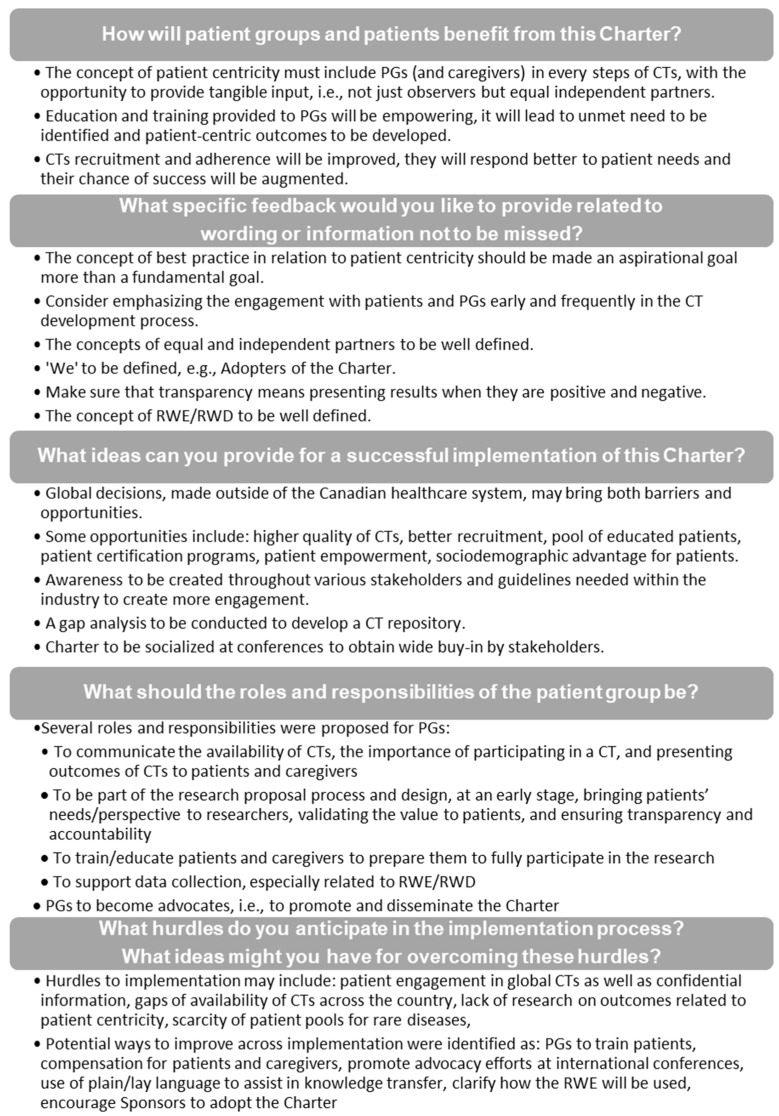
Summary of the feedback collected during the socialization of the draft Canadian Cancer Clinical Trials Stakeholder Charter, including the MVPs from the 2018 CCC conference. CCC: Colorectal Cancer Canada; CT, clinical trial; MVP: minimum viable product; PG, patient group; RWD, real-world data; RWE, real-world evidence.

Started from ground zero and inspired by the “Novartis’ Commitment to Patients and Caregivers” [48], the Charter, initiated by CCC, was developed by a dedicated WG through a rigorous process involving relevant stakeholders, including scientists/clinicians, individuals from PGs and advocacy groups, cooperative academic CT oncology groups, representatives from health agencies, research networks/consortium, industry partners, contract research organizations (CROs), as well as key participants from CCC. As a key contribution to the development of the Charter, stakeholders attending the CCC Patient Group Pathway Model to Accessing Cancer Clinical Trials and Real-World Evidence Methodologies Conference (Table S2) provided feedback, consolidated as minimal viable products (MVPs) in Figure 3, which was taken into consideration to finalize the Charter.

### 3.2. The Canadian Cancer Trials Stakeholder Charter

Adopters of the Charter believe that stakeholders must all strive for excellence when conducting cancer research and development as well as direct their efforts toward the development of treatments that provide clinically meaningful results with improved quality of life and patient outcomes.

The Charter—entitled the Canadian Cancer Trials Stakeholder Charter—was built around five guiding principles—1—patient centricity; 2—commitment to education and training; 3—collaboration as equal and independent partners in research; 4—transparency and accountability; and 5—high standards in data collection integrity and honesty [47].

Aiming to make CTs accessible to all cancer patients, improve the design and implementation of CTs, expend recruitment and retention of patients in CTs, and further advance cancer research and treatment, and empower patients and PGs to fulfil their role, the adopters commit to the Charter’s five tenets described below. A Charter’s glossary is provided in Table S3.

#### 3.2.1. Tenet 1: Making Patient Centricity a Norm in Clinical Trials

What it means:

Using our best efforts to understand and integrate the patient perspective throughout the CT continuum by considering their experiences and needs. Patient input will be considered throughout the entire CT continuum.

Commitment to:

- Ensure that studies are designed to realize outcomes that are relevant to patients and includes their preferences and trade-offs, achieve clinically meaningful results and enhance patient quality of life and health outcomes, while minimizing the burden of disease and treatment on patients;
- Increase access to CTs by reducing barriers and ensuring that eligibility criteria are fair and appropriate;
- Simplify the informed consent document to provide transparent, comprehensible CT information in a language that is relevant for patients;
- Engage patients in a two-way communication throughout the CT continuum (CTC), solicit and incorporate their feedback, and provide access to mechanisms, such as digital and mobile health technologies where possible;
- Connect patients to internal and external support programs and other resources through convenient and user-friendly channels; and
- Provide patients with uninterrupted access to CT therapies.

#### 3.2.2. Tenet 2: Supporting Education, Training and Development of Patient Group Members for Effective Participation in the Design and Implementation of Clinical Trial Protocols on Behalf of Patients

What it means:

Engaging PGs as equal partners, consistent with all other stakeholders, throughout the CT continuum and therefore recognizing their expertise, input, and experience are unique and valued.

Commitment to:

Engage with patients and PGs early on as well as on an ongoing basis to work together effectively throughout the CTC.

- Support training of PGs and evaluation initiatives while working collaboratively with them, encouraging their input and active participation in the development, implementation and reporting of a CT;
- Support PGs’ ability to record patient values and preferences both during and post CT;
- Support training of other research Stakeholders including the Sponsor representatives, to ensure best practices are met in their engagement with PGs; and
- Evaluate and share the impact of our engagement with PGs.

#### 3.2.3. Tenet 3: Collaborating with Patient Groups as Equal and Independent Partners to Optimize the Success of Clinical Trials

What it means:

Striving to change the culture of CTs to ensure that PGs are recognized as equal, independent partners to optimize the success of the trial.

Commitment to:

- Build strong partnerships with PGs and all Stakeholders by agreeing on joint expectations, responsibilities, and the commitment to promote co-operation;
- Include patient insight in the development of the consent process and patient-facing materials;
- Facilitate the connection between trial participants and PGs;
- Promote awareness and education of CTs among PGs, while integrating their involvement in the design and implementation of CTs;
- Work with PGs to integrate patient needs from the conception of CTs, to expedite and facilitate access to CT information, patient-facing materials and CT consent; and
- Act with integrity and respect the independence of PGs.

#### 3.2.4. Tenet 4: Adhering to Transparency and Accountability throughout the Clinical Trial Continuum

What it means:

Building mutually transparent and open communication with PGs on behalf of patients as part of the CT continuum and maintaining high levels of accountability.

Commitment to:

- Bolster trust through open dialogue and interaction with PGs and seeking their input throughout to ensure CT lifecycle and following the CT, as required;
- Work collaboratively with PGs to better understand and address patient unmet needs, preferences and trade-offs, the burden of current treatments and disease;
- Share information with PGs and patients in a neutral, uninfluencing and objective manner, where data is presented clearly and accurately as well as in a balanced and fair context, to allow PGs to form their own independent opinion and interpretation;
- Hold ourselves to highest levels of accountability by ensuring that the independence of all stakeholders involved is maintained and by implementing clear conflict of interest and disclosure guidelines;
- Develop trust and confidence in the methods used;
- Transparently share the aggregate results of CTs with patients and PGs, regardless of the trial outcome, in a timely, efficient and comprehensible manner; and
- Transparently share the individual results with the patient and/or patient guardian in the case of a pediatric study, regardless of the outcome, in a timely, efficient and comprehensible manner.

#### 3.2.5. Tenet 5: Maximizing the Potential to Collect and Utilize RWE/RWD Captured in All Clinical Trials

What it means:

Real-world evidence/real-world data (RWE/RWD) will be considered in all CTs to optimize the quality of evidence, minimize risk of bias and build upon public and stakeholder trust in research credibility and reliability.

Commitment to:

- Consider RWE/RWD in the collection of data in order to render the results more generalizable to achieve greater external validity, better support access, appropriateness of use and affordability of the therapeutic interventions being tested in CTs;
- Communicate (in lay language for good comprehension by PGs and patients), the research goals, methods, procedures, RWE/RWD collected as well as the findings resulting from the use and analysis of this data;
- Ensure that the RWE/RWD data is complete, reliable, and processed in a consistent manner. Best practices in data collection and analysis should be applied from the initiation of the trial study design and maintained throughout the CTC; and
- Share RWE/RWD data post CT in a timely manner to ensure the greatest impact of patients/caregivers to help with decision making.

## 4. Discussion

The International Association for Public Participation has defined five levels of engagement outlining the impact of partnership with patients in CTs, which are intrinsically conveyed in the Charter—inform, consult, involve, collaborate, and empower [2]. The Charter offers a unique and tangible framework for cancer CT stakeholders to commit to and to gauge their organizations’ patient centricity in CTs. The vision integrated into the Charter is that PGs would be empowered as equal partners in CTs, completely integrated throughout the CTC, from the conception through translation.

Aligned with the Charter tenets and commitments, recommendations from the CTTI/C-CTTI and other initiatives aiming at improving research co-design were to—build trust and report between researchers and co-design participants, use clear and consistent terminology, provide training if necessary, invest in co-design notably by allocating sufficient time and resources, empower and nurture participants to promote engagement, communicate and update all parties regularly, and report findings regardless of outcomes, to name a few [1,4,5,11,13,17,18,19,28,35,44].

With regards to encompassing the concept of patient centricity and to enhancing research collaboration with PGs, a recent review has identified the main reported co-design activities to be focus groups, interviews, and surveys [1]. Specifically for CTs, patient involvement in developing plain-language summaries has been identified in a global industry survey as most often implemented and piloted [26]. However, the authors also reported that more organizational patient-centric initiatives are in the planning stages than are being implemented or piloted.

In terms of limitations, Heckert et al. [5] reported that challenges the most often described by investigators and partners on engagement were three-fold—(1) infrastructure to support engagement, (2) building relationships, and (3) maintaining relationships. Challenges to both building and maintaining relationships were related to having authentic, positive interactions that facilitate mutual understanding, full participation, and genuine influence on the projects. On a broader scale, patient groups are an heterogenous entity, which should be considered in assessing the challenges of their engagement in the CT continuum [49].

As reported from a survey on 25 patient-centric initiatives within pharmaceutical and biotechnology companies [26], the primary barriers to the adoption of such initiatives were to gain internal company buy-in and authority to implement them. Findings from a similar survey revealed that PGs valued their contributions to research protocol development, funding acquisition, and interpretation of study results more highly than those contributions were valued by industry and academic respondents [50]. Acknowledged by du Plessis et al. [24] following discussion from a panel of senior pharmaceutical representatives, true patient centricity requires a change in the industry’s cultural mindset, an increase in public trust, clearer roles and responsibilities within pharmaceutical organizations, openness to learn from others, and a framework to measure success.

To succeed with patient engagement and perhaps to guide education and training, patient competencies identified in a recent review include an understanding of research as well as a desire to contribute to the team effort. Frisch et al. [3] further reported that several patient competencies may be learned on-the-job, and that it is not expected that all patients be able to interpret and evaluate the research, but to understand its purpose, to share their patient experience, and to show willingness to contribute to the dissemination of the study findings. Explicit in the Charter and conveyed in the stakeholder feedback received on the draft Charter is the importance of engaging patients and PGs early and regularly in the CTC, including for the collection of RWE/RWD [51]. This was reported as a key component to the success of the GO-CART patient engagement program in blood cancer as well as in research conducted in other areas, such as depression and asthma [4]. confirming findings from other initiatives [11,29,30].

Stakeholders from the CCC conferences have identified needs in several areas—1—clearly define PG and patient roles and responsibilities; 2—create a comprehensive communication strategy and plan; 3—build an advocacy plan; and 4—clearly define standardized metrics and measurements. Moving forward, as an aim to integrate the patient voice into the Canadian cancer CTC and using the recent CTTI recommendations on RWE/RWD [51], the next phases include the dissemination, adoption, and implementation of the Charter, the establishment of a PG training program, and the development of RWE/RWD methodologies.

## Supplementary Materials

The following are available online at https://www.mdpi.com/article/10.3390/curroncol28020137/s1, Table S1: Members of the Charter working group throughout the Charter development process, Table S2: Stakeholders attending the 2019 CCC PG Pathway Model to Accessing Cancer Clinical Trials & Real-World Evidence Methodologies Conference, Table S3: The Canadian Cancer Clinical Trials Stakeholder Charter’s Glossary.
