# Supplementary material for: Patient and Patient Group Engagement in Cancer Clinical Trials: A Stakeholder Charter
Source: Curr Oncol. 2021 Apr 8;28(2):1447–58. doi: 10.3390/curroncol28020137 (PMC8167642; doi:10.3390/curroncol28020137)
Supplement: Supplementary file 1 [file curroncol-28-00137-s001.pdf]

Supplementary Materials

Patient and Patient Group Engagement in Cancer Clinical Trials: A Stakeholder Charter

Stéphanie Michaud [1], Judy Needham [2], Stephen Sundquist [3], Dominique Johnson [4], Sabrina Hanna [5], Sharareh Hosseinzadeh [6], Vatche Bartekian [7], Patricia Steele [8], Sarita Benchimol [8], Nathalie Ross [9] and Barry D. Stein [8,*]

Table S1. Members of the Charter working group throughout the Charter development process.

Name	Affiliation
Karen Arts*	N2
Vatche Bartekian	Vantage BioTrials
Emanuela De Franco*	Novartis
Sabrina Hanna	The cancer collaborative
Sharareh Hosseinzadeh	Novartis Pharma Canada
Isabelle Jodoin*	Novartis Pharma Canada
Dominique Johnson	McPeak-Sirois Group for Clinical Research in Breast Cancer
Stéphanie Michaud	BioCanRx
Judy Needham	Canadian Cancer Trials Group
Dawn Richards*	Clinical Trials Ontario
Stephen Sundquist	Canadian Cancer Clinical Trials Network
Patricia Steele	Colorectal Cancer Canada
Sarita Benchimol	Colorectal Cancer Canada
Barry Stein	Colorectal Cancer Canada

The meetings were facilitated by Anne Marie Weight (Elements Strategy) with the assistance of Elle Doherty (CCC). *Previous members.

Table S2. Stakeholders attending the 2019 CCC PG Pathway Model to Accessing Cancer Clinical Trials & Real-World Evidence Methodologies Conference.

Name	Affiliation
**Patient groups**	
Niya Chari	Canadian Breast Cancer Network
Martine Elias	Myeloma Canada
Terry Hawrysh*	Patient partner from the GO-CART program
Cathie Jackson	Colorectal Cancer Canada
Dominique Johnson	McPeak-Sirois Group for Clinical Research in Breast Cancer
Nathalie Laplante	McPeak-Sirois Group for Clinical Research in Breast Cancer
May Karry	Colorectal Cancer Canada
Morgan Kennedy	Colorectal Cancer Canada
Katya Kruglova	Colorectal Cancer Canada
Jackie Manthorne	Canadian Cancer Survivor Network
Carole McMahon*	Former pCODR patient member
Anne-Marie Myers	Colorectal Cancer Canada
Judy Needham	Canadian Cancer Trials Group
Frank Pitman	Colorectal Cancer Canada
Zal Press	Patient Commando
Bunnie Schwartz	Colorectal Cancer Canada
Patricia Steele	Colorectal Cancer Canada
Barry Stein	Colorectal Cancer Canada*
Eva Villalba	Coalition Priorité Cancer
Chelsey Weir	Colorectal Cancer Canada
**Clinical research centres**	
Winson Cheung*	Tom Baker Cancer Centre
Manoj Lalu*	Ottawa Hospital Research Institute
Ingrid Saba	Brooks Life Sciences
Stephen Sundquist	3CTN
Sara Urowitz	Canadian Cancer Research Alliance

Philip Wong	CHUM
**Healthcare and research agencies**	
Sylvie Bouchard*	INESSS
Penny Chipman*	McGill University Health Center
Maxime Dumais	Oncopole
Melissa Hunt*	Health Canada
Stéphanie Michaud*	BioCanRx
Dawn Richards*	Clinical Trials Ontario
**Industry**	
Negin Ashki	Eli Lilly Canada
Jennifer Atkinson*	Roche Canada
Vatche Bartekian	Vantage BioTrials
Maude Beaulieu	Amgen
Nathalie Brazeau	Janssen
Grace Castillo-Soyao	Self Care Catalysts
Alexandra Chambers*	Novartis Oncology
Monique Deol*	Roche Canada
Jianmin Duan	Duan Pharmaceutical Consulting Inc.
Michael Duong*	Hoffmann-La Roche
Felicia Flowitt	AstraZeneca
Leigh Funston	AstraZeneca
Aman Garg	FLS transportation
Valerie Higenell*	Exactis
Chantal Lacasse	AbbVie
Daniel Lacroix	IQVIA
Stephanie Lacroix	Merck Canada Inc.
Frédéric Lavoie*	Pfizer
Johanna Mancini*	IQVIA
Suzan McNamara	Exactis Innovation
Charles Milliard	National Cabinet
Christine Montgrain	AbbVie
Wendy Morton	Merck Canada
Albert Nguyen	IQVIA
Josée Pelletier	BMS
Francois Peloquin	Pfizer Canada ULC
Sabrina Perri	Novartis Canada
Marie Prévost	AbbVie
Vincent Raymond	Pfizer Canada
Philippe Renaud	Bayer
Ugendhar Surkanti	Onco Pharma
Helen Trifonopoulos*	Novartis Oncology
Brigitte Viel	Pfizer Canada
Diane Wright*	Roche Canada
**Key experts**	
Nathalie Ross	Nathalie Ross, Ph.D., MWC
Anne Marie Wright*	Elements Strategy Inc.

*Meeting presenter, moderator, and/or facilitator.

Table S3. The Canadian Cancer Clinical Trials Stakeholder Charter's Glossary.

Term/Expression	Definition
**Adopters of the Charter:**	A person or an organization/institution that chooses to take up, follow or use the Charter for its intended purpose.
**Clinical Trial:**	Any oncology investigation in human subjects intended to determine the clinical pharmacological, pharmacokinetics, and/or other pharmacodynamics effect of an investigational agent, and/or to identify any adverse reactions to an investigational agent to assess the agent's safety and efficacy.
**Clinical Trial Continuum:**	The sequential series of steps involved in the development of clinical trials. These include 1- the Discovery and preclinical stages, the Phase 1-3 trials, the Regulatory review process and the post-approval process.
**Clinical Trial Lifecycle:**	The sequential series of steps involved in the development of clinical trials. These include the discovery and preclinical stages, as well as Phase 0 trials to learn how an agent is processed in the human body and how it may affect the body, Phase I trials seek to find the best dose of a new agent and assess overall safety, Phase II trials aim to further assess the safety as well as determine if the agent works, Phase III trials further investigate product safety, effectiveness of a new agent as compared to the standard of care in larger study populations, Phase IV trials, otherwise known as post-marketing trials, serve to test agents previously approved for use by Health Canada (agents include drugs).
**Consent Process:**	A process in which a healthcare provider educates a Patient about the risks, benefits and alternatives of a given procedure, intervention or treatment. The Patient must be competent to make a voluntary decision about whether to undergo the said procedure or treatment.
**Patient (Cancer Patient):**	A person who is receiving medical treatment for a malignant growth or tumour.
**Patient Centricity:**	The process of designing a service or solution around the Patient. In Clinical Trials it is a trial designed with the Patient at the forefront to improve the overall experience for the Patient by including their concerns and priorities in the design ensuring that the Clinical Trial answers specific unmet needs of the Patient.
**Patient-Facing Materials:**	Documentation associated with each phase of a clinical trial that is written in a language that is tailored to the audience and their cultural area. Titles of studies, terminology and wordings are translated in a patient-friendly manner to ensure maximum transparency and comprehension.
**Patient Group:**	A term encompassing patient advocacy organizations, disease advocacy organizations, voluntary health services, non-profit research foundations and public health organizations for clarity of focus. Our use of the term Patient Group in not meant to refer to individual Patients or individual advocates.
**Patient Preferences/ Perspectives:**	Patient Preferences/Perspectives related to therapies and outcomes regarding willingness to accept uncertainty and trade-offs based on potential harm versus benefits. Benefits-risk assessment may also seek to identify subgroups of Patients in a heterogeneous population based on preferences.
**Protocol (Clinical Trial Protocol):**	Every clinical investigation begins with the development of a clinical trial protocol. The protocol is a document that describes how a clinical trial will be conducted (the objective(s), design, methodology, statistical considerations and organization of a clinical trial), and ensures the safety of the trial subjects (participants) and integrity of the data collected (source: Clinical Research Resource Hub).
**Real-World Data (RWD):**	Real world data are the data relating to patient health status and/or the delivery of health care routinely collected from a variety of sources, such as electronic health records and product and disease registries (source: Food and Drug Association (FDA)).
**Real-World Evidence (RWE):**	Clinical evidence regarding the usage and potential benefits or risks of a medical product derived from analysis of RWD.
**Researchers:**	Individuals engaged in the conduct of scientific research at an academic or pharmaceutical institution.
**Stakeholders:**	Parties with concerns or interests in an organization, endeavor, or initiative. Stakeholders include but are not limited to:<br>• Governmental institutions and agencies<br>• Medical Researchers<br>• Patients/Patient Groups<br>• Pharmaceutical/Biotech Companies<br>Regulatory bodies (internal/external)
**Standard of Care Treatment:**	Treatment that is accepted by medical experts as an appropriate treatment for a certain type of disease and that is widely used by healthcare professionals. Also called best practice, standard medical care, and standard therapy (source: National Cancer Institute (NCI)).
**Standard of Work Practice:**	The regulations, guidance and industry standards that make up Standard of Work Practice of good clinical practice are intended to provide assurance that the rights, safety and well-being of Clinical Trial subjects are protected.
**Study Design:**	Study design is a particular framework, or the set of methods and procedures used to collect and analyze data on variables specified in a particular research problem (source: National Institutes of Health (NIH)).
**Therapeutic Intervention (Oncology):** A	clinical effort to improve the well-being of someone who has cancer. Therapeutic interventions in oncology include chemotherapy, immunotherapy, and surgery.
**Trial Information Data:**	The computerized form of results and analysis obtained throughout the Clinical Trial process, including Patient treatment responses and other derived variable.
